# Cutaneous melanoma frequencies and seasonal trend in 20 years of observation of a population characterised by excessive sun exposure

**DOI:** 10.1515/raon-2015-0039

**Published:** 2015-11-27

**Authors:** Serena Bonin, Antonio Albano, Nicola di Meo, Alessandro Gatti, Giuseppe Stinco, Fabrizio Zanconati, Giusto Trevisan

**Affiliations:** 1 Department of Medical Sciences, Unit of Dermatology, University of Trieste, Trieste, Italy; 2 Unit of Dermatology; Azienda Ospedaliero-Universitaria Ospedali Riuniti di Trieste; Ospedale Maggiore, Trieste, Italy; 3 Department of Experimental and Clinical Medicine, Institute of Dermatology, University of Udine, Italy; 4 Department of Medical Sciences, Unit of Surgical Pathology, University of Trieste, Trieste, Italy

**Keywords:** cutaneous melanoma, sun exposure, frequencies, multiple melanomas, gender related differences

## Abstract

**Background:**

Cutaneous melanoma is an aggressive form of skin cancer. It has become an increasingly common neoplasm in the most developed countries, especially among individuals of European origin.

**Patients and methods.:**

Anonymous data of patients with cutaneous melanoma were collected from the diagnostic database of the University Hospital of Trieste from 1 January 1990 to 10 December 2013. Our study is based on a population which was constant over the period of observation; it was also well-defined and characterised by unrestrained sun exposure.

**Results:**

The number of cutaneous melanomas increased during the period of observation with a seasonality trend and gender related differences both for anatomical sites distribution and stage of the disease. Moreover, 6% of our cohort developed multiple melanomas.

**Conclusions:**

In a well-defined population devoted to excessive sun exposure the frequencies of skin melanomas roughly doubled from 1990 to 2013 following a seasonal trend. In that population, prevention efforts according to gender specific risk behaviour, as well as follow-up programmes both for evaluation of metastatic spreading and for early diagnosis of additional skin melanomas, are crucial due to gender specific differences and to the occurrence of multiple melanomas.

## Introduction

Cutaneous melanoma has become an increasingly common neoplasm in most developed countries, especially among individuals of European origin.^1^,^2^ Different patterns referred to patients’ sex and age have been observed worldwide; most recent estimates indicate wide North–South and East–West variation of melanoma incidence in Europe, with the lowest rates in Southern and Eastern countries.^3^

The main possible reason for the general increasing melanoma incidence over the last 40 years is greater exposure of pale Caucasian skin to natural ultraviolet (UV) radiation.^2^ Epidemiological studies suggest a relationship between suntan habits and high risk of melanoma. Sun exposure is highly prevalent in all age groups, especially among the young; it is influenced by certain convictions and attitudes towards suntan, and it is stimulated by peer pressure and beauty reasons. Although the general public is now aware that sunlight exposure has been leading to increased risk of skin cancer over the decades, most people still believe that a tanned person looks healthier.^4^ Moreover, also environmental risks referred to climate changes^5^ and increased reporting of *in situ* melanomas^6^ seem to contribute to the rise of melanoma rates.

The aim of this study is to analyse the frequencies and characterise melanoma patients in one of the Italian cities with the highest incidence of cutaneous melanoma^7^ and whose inhabitants sunbathe excessively.

## Patients and methods

Anonymous data from patients with cutaneous melanoma were collected from the diagnostic database of the University Hospital of Trieste. Inclusion criteria were a diagnosis of cutaneous melanoma and to be resident in the Italian province of Trieste. Data include gender, age at diagnosis, date of diagnosis and anatomical site of melanoma onset. Data were collected separately for *in situ* and invasive melanoma from 1 January 1990 to 10 December 2013. For patients submitted to BRAF mutation analysis for therapeutic issues also BRAF mutational status was retained. For survival and analyses related to melanoma thickness, histological type and disease progression, we retained those patients who were followed up by the Dermatology unit of the University Hospital of Cattinara. Clinical data criteria as well as results are reported in the Supplementary file. The study was conducted according to the Declaration of Helsinki protocols. Moreover, clinical data were available only for patients who signed an informed consent for research use of their data.

### Statistical analyses

The distribution of the clinical, histological, and epidemiological categorical variables was compared by the chi-squared test. ANOVA and t-test were performed for continuous variables depending on the number of categories. Seasonality test was evaluated by means of the Walter and Elwood test.^8^ To estimate trend across groups for non-parametric data the Cuzick’s test was used. All p-values are two-sided with values <0.05 regarded as statistically significant. Statistical analyses were performed with the Stata/SE 12 package (Stata, College Station, TX).

## Results

### Frequencies

**FIGURE 1.**
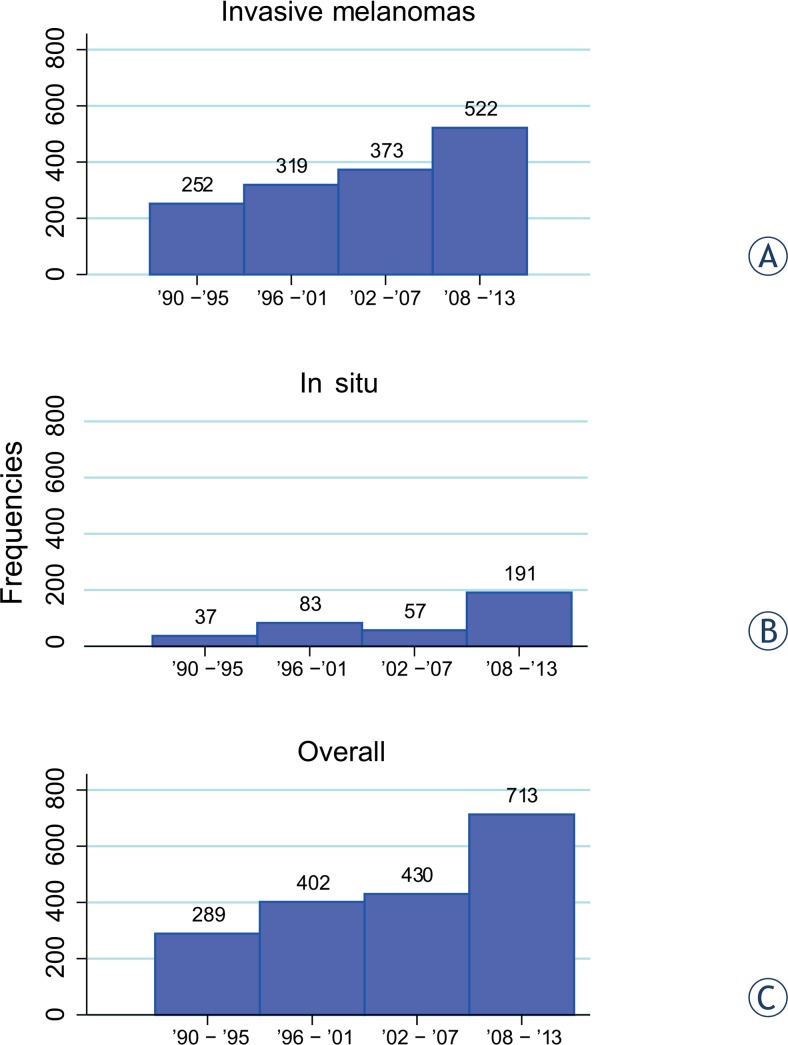
Frequencies of cutaneous melanoma over years of observation: **(A)** invasive melanomas; **(B)** *in situ* melanomas; **(C)** invasive and *in situ* melanomas.

From 1990 to 2013, 1834 patients had a diagnosis of cutaneous melanoma. They were all Caucasian residents of the province of Trieste between 14 and 98 years of age. Data on *in situ* as well as invasive cutaneous melanoma by age group and gender are reported in Table 1. Overall, mean age at diagnosis was 59 years (± 16.3 years) with 50% gender distribution. Age at diagnosis for men was significantly higher (average age 62 years) than for women (average age 57 years) (p < 0.001). The number of melanoma patients has increased over the years both for *in situ* and invasive melanomas and that number was more pronounced in the last 6 years (Figure 1). The diagnosis of melanoma was more frequent for middle-aged and older patients, although an increment for younger patients (aged < 40) over years has been observed.

**FIGURE 2.**
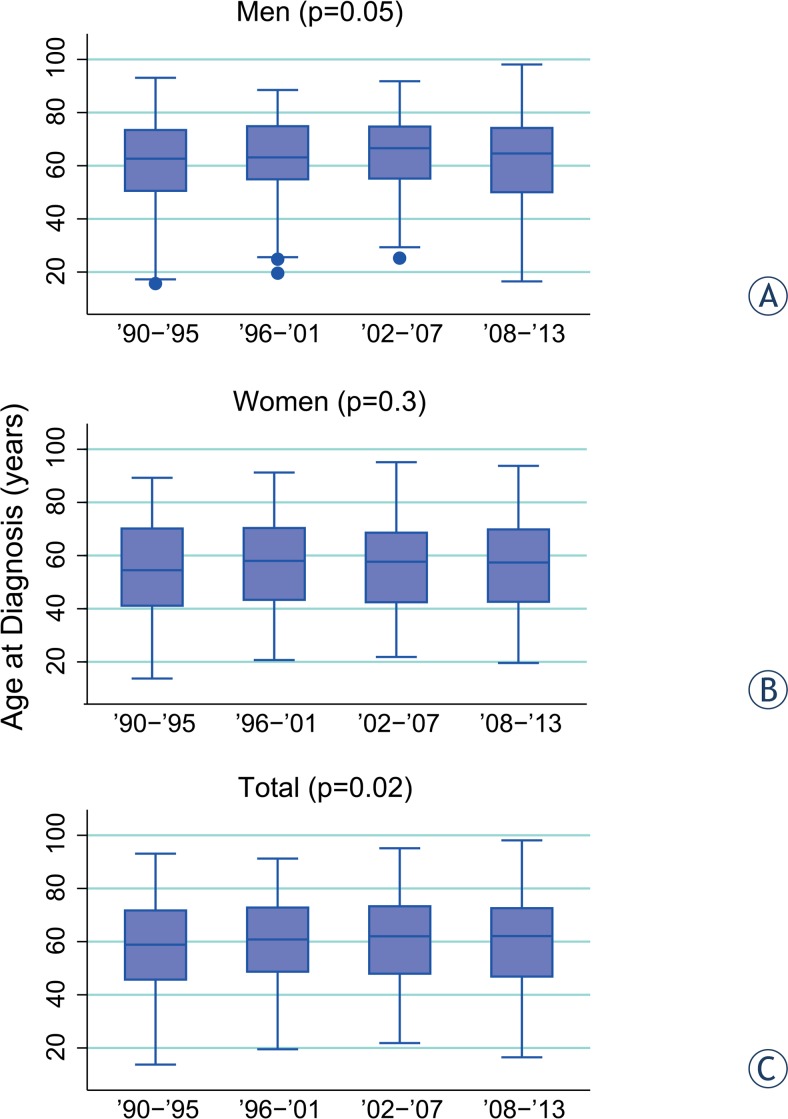
Age at diagnosis over years of observation: **(A)** men; **(B)** women; **(C)** total.

When we divided the 24 years of observation into 4 periods of 6 years, we did not find any variation of age at diagnosis over the years (p = 0.1), even by sex stratification (p = 0.06 for men and p = 0.5 for women). However for invasive melanomas a significant increment of age at diagnosis was observed over the 4 intervals of observation (p = 0.02) (Figure 2). This information was maintained for men (p = 0.05), but not for women (p = 0.3) (Figure 2). Age at diagnosis for men increased significantly from 61 (± 18 years) years to 64 (± 15 years) years. Age at diagnosis for *in situ* melanomas did not vary across years (p = 0.3), for both genders (p = 0.6 for men and p = 0.4 for women).

### Anatomical sites

The distribution of primary cutaneous melanomas by anatomical sites is reported in detail in Table 2. Hereafter only significant results are reported. Overall, melanomas of the trunk were more frequent in men (54%) than women (35%) (p < 0.001). This result was maintained considering separately invasive and *in situ* melanomas (p < 0.001 for both); therefore 53% of men and 36% of women developed an invasive melanoma of the trunk. With regard to *in situ* neoplasm the proportion of melanoma of the trunk was 60% for men and 32% for women. Melanomas of the head and neck were also more frequent in men (p = 0.003), who had that localization in 12% of cases compared to 8% of women. The distribution of invasive melanomas of head and neck was different between genders (p = 0.02), although it was comparable for *in situ* (p = 0.07).

The percentage of melanomas of the lower limbs was higher in women (29% *vs.* 10%) (p < 0.001), both for invasive melanomas (p < 0.001) and *in situ* ones (p < 0.001) (Table 2).

Invasive melanomas of hands and foot were more frequent in women (p = 0.05) with a proportion of 4.4% of cases in comparison to 2.5% of men; this observation was not confirmed for *in situ* melanoma (p = 0.7).

When we divided the 24 years of observation into 4 periods of 6 years the proportions of melanomas by anatomical sites remained essentially the same (p = 0.1), both for men (p = 0.2) and women (p = 0.4).

### Seasonality

**FIGURE 3.**
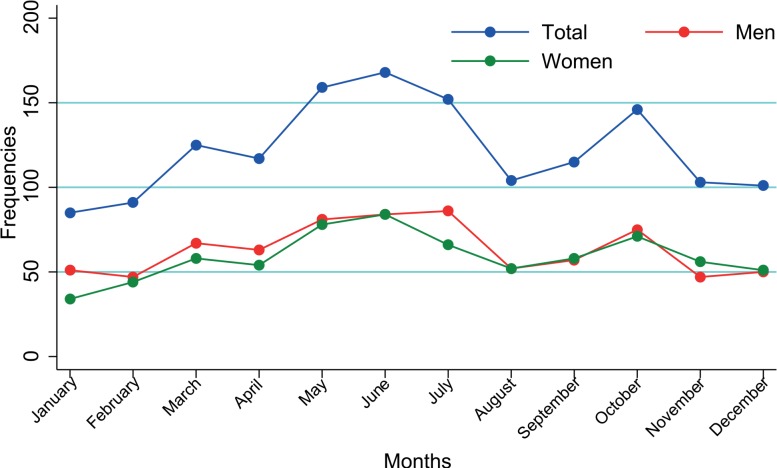
Seasonality of invasive cutaneous melanoma.

The overall monthly diagnosis of invasive melanomas showed significant excess from the cyclic variation with the maximum around the month of June (represented by θ = 171°) and another lower peak in October (p < 0.001) (Figure 3). The same pattern was present for males (θ = 162° - early June, p < 0.001) and females (θ = 181°- late June, p = 0.0002) (Figure 3). No statistically significant cyclic trend was evidenced for *in situ* melanomas (p = 0.2), either for men (p = 0.5) or women (p = 0.3).

According to the anatomical site of invasive melanoma onset, head and neck did not show cyclic trend (p=0.3), but all the other sites did so: invasive melanomas of the hands and foot peaked in late May (θ = 147°, p = 0.002), those that were developed on the trunk and lower limbs peaked in June (θ = 157°, p = 0.03; θ = 178°, p = 0.0001, respectively) and invasive melanomas of the upper limbs peaked in July (θ = 205°, p = 0.004).

### Multiple melanomas

In our cohort 112 patients developed multiple melanomas: 26 of them had synchronous melanomas and 86 had metachronous melanomas.

#### Synchronous

With respect to synchronous melanomas all patients, except one, had diagnosis of *in situ* melanomas for both neoplasms.

There was no difference between genders for age at diagnosis. Distribution of anatomical sites was significantly different between genders (p = 0.04), mainly because melanomas of the lower limbs were more frequent among women (p = 0.03; 21% *vs.* 0); the other anatomical sites were similarly distributed between genders (p = 0.3). No patient belonging to this group of multiple melanomas developed the neoplasm on hands and foot.

#### Metachronous

**FIGURE 4.**
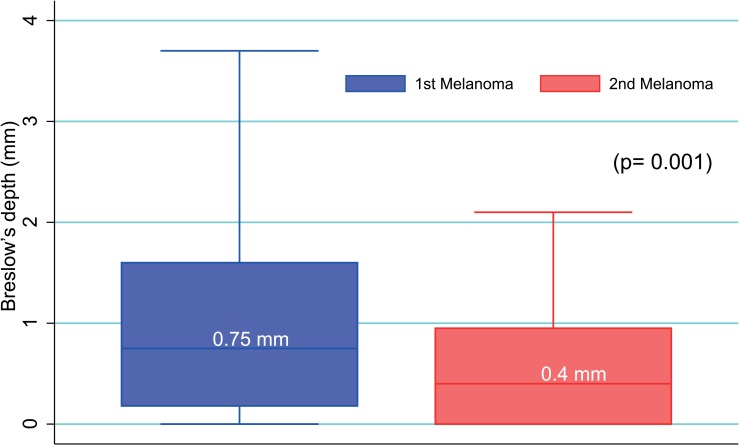
Box plot representing the Breslow’s depth variation between 1^st^ and 2^nd^ cutaneous melanoma in patients with metachronous melanomas. Median values are reported in boxes

Of the 86 patients who developed metachronous melanomas 53 were males and 33 were females, therefore men developed multiple metachronous melanomas (p = 0.02) more frequently. The appearance of the second melanoma did not follow any seasonal trend (p = 0.07). Most patients had a diagnosis of invasive melanomas for both neoplasms. On average, the second primary melanoma developed 3.7 years after the first one (min-max = 0–17 years), without any significant difference in time of appearance among genders (p = 0.8) and anatomical sites of the primary and secondary melanomas (p = 0.8 and p = 0.5, respectively). Overall, the location of primary melanomas was not significantly different between genders (p = 0.07), although a higher frequency of primary melanomas of the trunk was observed for men (62% *vs*. 33%, p = 0.02) and a higher frequency of primary melanoma of the lower limbs was observed in women (24% *vs*. 9%, p = 0.05) in agreement with data obtained from the entire cohort. In 30 patients (35%) the second melanoma developed in the same anatomical region as the first one, more frequently on the back (40%) and lower limbs (23%). The site of second primary melanomas differed between genders (p = 0.03) with a prevalence of the melanoma of the trunk (62% *vs*. 36%; p = 0.01) for men and lower limbs for women (27% *vs*. 4%; p = 0.002). Breslow’s depth of primary melanomas was significantly higher than that of secondary melanomas (p = 0.001) (Figure 4). Similarly, the mean number of mitoses of primary melanomas was higher than that of the second ones (1.5 *vs*. 0.6, p = 0.01) and on average the stage of the first lesion was also higher than the second one (p = 0.01).

### BRAF mutational status:

BRAF mutational status was assessed in 40 patients for therapeutic issues due to disease progression. Of those 25 had mutations at BRAF gene while 15 were wild type (see supplementary file for details). Patients with mutant BRAF gene were significantly younger (average age 52 years) than those with wild type BRAF melanoma (average age 64 years; p = 0.03), in particular this observation was confirmed in females (p = 0.04), but not in males (p = 0.2) as shown in supplementary Table 2.

BRAF mutational status was also related to anatomical sites (p = 0.03): in our sub-group of patients BRAF mutations prevail in melanoma of trunk compared to melanoma of the hand and foot that were all wild type for BRAF.

### Clinical data and cancer specific survival

Elaboration of clinical data and cancer specific survival did not add anything new to cutaneous melanoma research, because they confirm already published evidence. Therefore, they are reported in the supplementary file.

## Discussion

This is a population-based study referred on data collection from cutaneous melanoma patients resident in Trieste, a seaside town of about 250.000 residents in North-eastern Italy. This population is stable and well-defined, and it was constant over the years of observation.^9^ Residents in this area are mainly fair skinned and blue eyes because of their Celtic.^10^ and Austro-Hungarian origins. The peculiarity of the inhabitants of this town referred to cutaneous melanoma is their unrestrained sun exposure, mainly for traditional and cultural reasons. For that particular reason any increment in cutaneous melanoma frequencies over the years is mainly due to environmental changes and suntan habits. The frequencies of cutaneous melanoma retrieved in this study are in agreement with the data reported by the Cancer registry of Friuli-Venezia Giulia region.^11^ The number of cutaneous melanoma increased during the period of observation, showing higher rates particularly for middle-aged and for elderly residents, who probably had not used any sun protection in their childhood and adult life, as reported by other authors.^12^ During our period of observation, a steep increment in cutaneous melanoma cases occurred; in addition in the last six years cutaneous melanoma frequencies doubled in comparison to 1990–1995 in a homogeneous and stable population. That increment may be due in part to improved registration of melanoma as well as to over-diagnosis^12^, but it’s unlikely that the frequency growth could be solely ascribed to those factors. Although there are no data supporting this hypothesis, a possible explanation could be the cumulative effect of air pollution and sun exposure, since the air pollution index has been correlated with skin cancer.^13^ Nonetheless, other individual factors such as the use of artificial sunbed, cosmetics including sunscreen, photosensitising drugs, and exogenous hormones could be additional risk factors for the development of cutaneous melanoma.^14^

In our cohort seasonality of cutaneous melanoma diagnosis was detected with a higher peak in June probably as a consequence of increased patient awareness and self-detection of suspected lesions due to summer clothing.^15^ The presence of a lower seasonality peak in October could find a reasonable possible explanation in the effect of intense sun exposure on the visibility of melanocytic proliferation after intense ultraviolet exposure. Consequently, in summer the highlighted pigmented lesions may alert the patients themselves or the physician.^15^ No seasonality has been detected for *in situ* lesions in agreement with Asken *et al*. as different explanations seem to work for invasive and *in situ* melanomas.^12^

Six percent of our cohort developed multiple melanomas, which are not uncommon in cutaneous melanoma patients.^16^ In agreement with Savoia *et al*. in our series of patients there are no differences in clinical characteristics or histopathological features of the first cutaneous melanoma between patients with single or multiple metachronous melanomas; the distribution of the first melanoma sites also follows the same pattern as single melanomas.^16^ The significant decrease in the mean Breslow’s thickness as well as in the number of mitoses and stage for the second metachronous melanoma is mainly due to the follow-up in those patients and to their increased awareness of pigmented lesion after having a melanoma.^16^ Even for early melanoma lesions the importance of scheduled and well defined follow-up procedures was stressed.

Regarding melanomas mutated at BRAF gene we observed a correlation between BRAF mutations and being young at the time of primary melanoma diagnosis in agreement with other authors^17^, and our observation was confirmed particularly in women. BRAF mutations seem to be associated also to anatomical sites of the primary lesion, with melanomas on the trunk presenting higher rate of mutations at BRAF gene, as already shown.^18^

Gender-related differences in the anatomical distribution and stage of cutaneous melanoma were found in our cohort. At diagnosis women present with thinner lesions and show up before men as shown by the lower age at diagnosis and lower Breslow’s depth for female (Results in Supplementary file). Women show *in situ* or stage I lesions, while men have stage II and locally advanced cutaneous melanoma as shown by an Austrian report which found similar results.^19^ Moreover, as already pointed out^20^, cutaneous melanoma predominated at lower limb and hand-foot for women and trunk for men. In our cohort invasive melanomas of head and neck were significantly more frequent in men as observed in England after the early 1990s.^21^ In agreement with others22 men also tended to develop multiple metachronous melanomas more frequently.

Overall those observations underline that prevention efforts should be increased taking into account gender-specific risk behaviour. As it was shown in the female population, less aggressive cutaneous melanoma are most likely diagnosed due to prevention.

## Conclusions

In a well-defined population excessively exposing to sunlight the frequencies of cutaneous melanoma have roughly doubled from 1990 to 2013. In that population gender specific differences as well as a seasonality trend have been observed, stressing the importance of prevention efforts taking into account gender-specific risk behaviour.

The fact that multiple melanomas are not really uncommon, especially in men, highlights the need for follow-up programmes not only for evaluation of metastatic spread but also for early diagnosis of additional cutaneous melanoma.

## Supplementary File



## Figures and Tables

**TABLE 1.** Frequencies by age range

**Calendar period of diagnosis**	**Age group (years)**							
***In situ***		*20–29*	*30–39*	*40–49*	*50–59*	*60–69*	*70–79*	*80+*
**Women**								
1990–1995	0	3	4	6	5	1	4	1
1996–2001	0	2	8	13	7	11	9	1
2002–2007	0	1	8	12	5	10	3	1
2008–2013	1	9	16	25	17	13	11	4
**Men**								
1990–1995	0	0	0	3	6	0	4	0
1996–2001	1	1	1	4	9	4	11	1
2002–2007	0	0	2	5	2	5	2	1
2008–2013	0	1	13	22	19	16	21	3
**Total *in situ***	2	17	68	90	70	60	65	12
**Invasive**	*0–19*	*20–29*	*30–39*	*40–49*	*50–59*	*60–69*	*70–79*	*80+*
**Women**								
1990–1995	4	10	14	17	28	21	20	13
1996–2001	0	11	20	19	36	31	29	17
2002–2007	0	9	21	35	30	35	24	23
2008–2013	0	7	28	38	36	62	39	29
**Men**								
1990–1995	3	5	11	12	21	30	26	17
1996–2001	0	3	10	16	35	32	40	20
2002–2007	0	2	8	21	35	54	42	34
2008–2013	1	6	16	34	39	71	78	38
**Total invasive**	8	53	112	192	260	336	298	191
**Overall (%)**	10 (1)	70 (4)	180 (10)	282 (15)	330 (18)	396 (21)	363 (20)	203 (11)

**TABLE 2.** Distribution of melanomas by sex and anatomical sites, percentage is included in brackets

**Anatomical sites**	**Female, n (%)**	**Male n (%)**	**Total n (%)**
***In situ* melanoma**			
Head and neck	10 (4.7)	15 (9.6)	25 (6.8)
Hands and feet	2 (1.0)	2 (1.3)	4 (1.1)
Back	21 (10.0)	35 (22.3)	56 (15.2)
Chest	48 (22.7)	60 (38.2)	108 (29.3)
Trunk (back+chest)	69 (32.7)	95 (60.5)	164 (44.5)
Upper limbs	50 (23.7)	20 (12.7)	70 (19.0)
Lower limbs	64 (30.3)	18 (11.5)	82 (22.3)
NOS	16 (7.6)	7 (4.5)	23 (6.3)
Total	211 (100)	157 (100)	368 (100)
**Invasive melanoma**			
Head and neck	60 (8.5)	92 (12.1)	152 (10.4)
Hands and feet	31 (4.4)	19 (2.5)	50 (3.4)
Back	86 (12.2)	156 (20.5)	242 (16.5)
Chest	165 (23.4)	244 (32.1)	409 (27.9)
Trunk (back+chest)	251 (35.6)	400 (52.6)	651 (44.4)
Upper limbs	107 (15.2)	125 (16.4)	232 (15.8)
Lower limbs	201 (28.5)	74 (9.7)	275 (18.8)
Other	2 (0.3)	0 (0.0)	2 (0.1)
NOS	54 (7.6)	50 (6.6)	104 (7.1)
Total	706 (100)	760 (100)	1466 (100)

NOS = not otherwise specified
