# Supplementary material for: Cutaneous melanoma frequencies and seasonal trend in 20 years of observation of a population characterised by excessive sun exposure
Source: Radiol Oncol. 2015 Nov 27;49(4):379–85. doi: 10.1515/raon-2015-0039 (PMC4722929; doi:10.1515/raon-2015-0039)
Supplement: Supplementary file 1 [file RADO-49-04-379_supp1.pdf]

Supplementary file

Cutaneous melanoma frequencies and seasonal trend in 20 years of observation of a population characterised by excessive sun exposure

Serena Bonin, Antonio Albano, Nicola di Meo, Alessandro Gatti, Giuseppe Stinco, Fabrizio Zanconati, Giusto Trevisan

Patient's method for Clinical Data

For survival and analyses related to melanoma thickness, histological type and disease progression we retained those patients followed up by the Dermatology unit of the University Hospital of Cattinara.

For those patients in addition to the previous data the followings were collected: follow-up period, histological type, thickness according to Breslow, mitotic index, the presence of ulceration, the analysis of SLN (sentinel lymph node), melanoma stage, the development of later recurrences with the anatomical site for metastases and the development of other primary melanomas. For survival analyses patients diagnosed up to 2010 were included so that a minimum observation period of three years is guaranteed. Therefore, excluding in situ melanomas, survivals analyses were made on a cohort of 655 patients.

The primary end-point of the study was cancer specific survival (CSS), which was defined, as the time from surgery to melanoma specific death. In detail, in this study disease free patients were: 1) those who were alive at the end of the follow-up (30/10/2013) or at the time of the last dermatological visit (if it was dated 2013); 2) those who died of causes not related to melanoma (end of follow-up is the death date); 3) those who were alive, but emigrated (end of follow-up is the emigration date). The log-rank test was used to check the dependence of patients' survival on single variables or on combinations of variables. To estimate the joint effects of the analysed covariates on patients' survival, the data were analysed by fitting the Cox proportional hazard regression model. Cox proportional hazard analysis included those variables which influenced CSS by log-rank test. As post-estimation for the proportional hazards assumption the Grambsch and Therneau test for Schoenfeld residuals was run.[1]

Methods for BRAF analyses

BRAF mutational status has been performed since 2010. At the beginning Sanger sequencing of PCR amplicon (protocol which successfully passed the UK NEQAS) was used as method during 2010 and 2011. Later the Therascreen BRAF RSQ PCR kit was used to detect V600E/Ec, V600D, V600K and V600R mutations at BRAF gene.

Clinical data

Of the 1278 patients followed up by the Dermatology unit of the University of Trieste 969 were retained, because they were resident in the Italian province of Trieste and because their diagnosis was made between 1990 and 2013. Of those first primary melanomas 205 were in situ and 764 invasive. Median follow-up of patients was 5.8 years (range 0-23 years). Median Breslow's depth was 0.9 mm and it was significantly different between genders: women, indeed, presented at diagnosis with thinner melanomas than men (1.28 mm vs 1.81 mm, p=0.001). Although non-significant, the average Breslow's thickness in patients who later developed other melanomas (metachronous) was lower if compared to patients with single melanomas (1.56 mm vs 1.00 mm including in situ; 2.0 mm vs 1.34 excluding in situ CM). In situ (stage 0) and local melanomas of stage I were more common in women, while men rather presented with stage II or regional diffused lesions (stage III) (p=0.004).

Excluding in situ melanomas, histological type of CM (Cutaneous Melanoma) was included in clinical records for 476 patients (68%). According

SUPPLEMENTARY TABLE 1. Cancer specific survival analysis results

Variables	HR	P (multivariate)	CI	P (Logrank)
Age at Diagnosis	1.03	0.05	1.00-1.06	0.0002[a]
Histotypes	1.23	0.02	1.03-1.47	<0.001
Breslow's depth	1.07	0.02	1.01-1.12	<0.001[b]
Ulceration	2.38	0.02	1.12-5.05	<0.001
Mitotic index	1.11	0.01	1.02-1.21	0.0002[c]
Anatomical sites	0.85	0.1	0.70-1.05	0.04

SUPPLEMENTARY TABLE 2. Results on BRAF mutational analyses

Variables	Wild Type	BRAF Mutated	p
Gender			
Men	8	15 (13 V600E and 4 V600K)	0.7
Women	7	10 (9 V600E and 1 T99-V600insT)	
Age at diagnosis	64	52	0.02
Men	68	57	0.1
Women	59	44	0.04
Anatomical sites			
Head and neck	3	4	
Foot	4	0	0.03
Trunk	4	15	
Limbs	4	6	

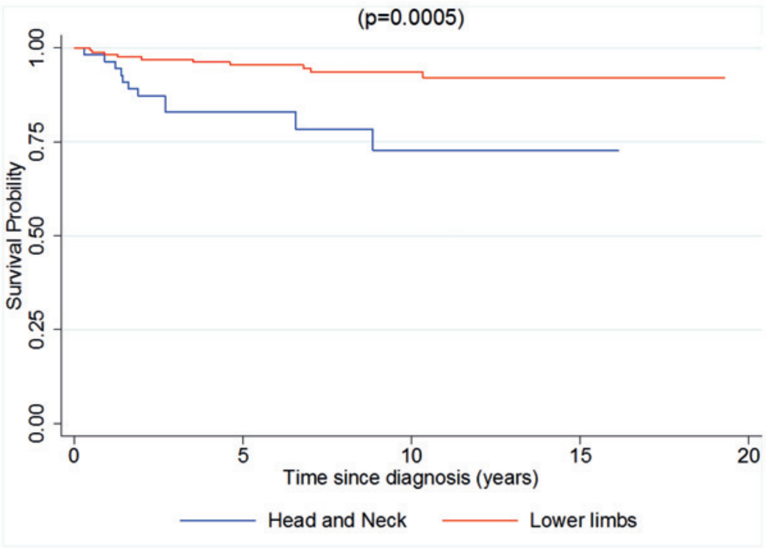

SUPPLEMENTARY FIGURE 1. Kaplan Mayer curve for overall survival of patients with head and neck and lower limbs melanomas

to pathological reports they were divided into 4 groups: lentigo maligna melanoma (LM), superficial spreading melanoma (SSM), nodular melanoma and Spitzoid melanomas. Acral and desmoplastic melanomas were recorded, but being few they were excluded from the statistical analyses and graphical representations. Age at diagnosis differed significantly among histotypes (p< 0.001); LM was diagnosed in older patients (median age 72 y) in comparison to SSM, nodular (median age 61 y for both) and Spitzoid melanomas (median age 47 years).

The histological type of CM was not different between genders (p=0.2). The distribution of Breslow's thickness as well as the mitotic index was significantly different among histotypes (p< 0.001 for both), notably SSM and Spitzoid melanomas were significantly thinner than nodular melanomas; median Breslow's thickness of nodular melanomas was 2.5 mm in comparison to 0.7 mm of SSM and 0.9 of Spitzoid melanomas. Moreover, nodular melanomas had on average more mitoses, counting on average 3 mitoses in comparison to SSM, Spitzoid (1 mitosis for both) and LM (0.1 mitosis).

Excluding in situ melanomas, the presence of ulceration was associated to Breslow's thickness (p<0.001), higher mitotic index (p<0.001) and to nodular melanomas. Notably thicker melanomas were ulcerated (mean Breslow's depth for ulcerated CM was 4.4 mm vs 1.5 mm in non-ulcerated CM) as well as with more mitoses and basically nodular melanomas were more frequently ulcerated if compared to the other histotypes (p< 0.001). In addition ulcerated melanomas had on average a significantly higher number of mitoses (1-2 mitoses $/mm^2$ vs 4 mitoses $/mm^2$ p<0.001).

Eventually, as a logical consequence also the stage at diagnosis varies among histotypes (p<0.001), therefore Spitzoid, LM and SSM were mostly of stage I and nodular melanomas of stage II.

Overall survival

Of the patients analysed for overall survival 20 were lost at follow-up, presumably for emigration. Excluding in situ melanomas and patients with a diagnosis later than 31st December 2010, 60 patients died from CM, 82 patients died of diseases unrelated to CM and 499 patients were alive. Significant results of overall survival analysis are summarized in Supplementary Table 1. Cox proportional hazard regression model was run including as covariates all variables which gave a significant result at Logrank test. Among the covariates stage was not included because it was split into its components. Among the analysed variables, gender did not affect patients' survival (p=0.2). Cutaneous melanomas developing at different body sites were associated with distinct patterns of survival outcomes (p=0.01): there was a higher rate of fail-

ures for patients with melanoma of the head and neck, while the opposite has been observed when melanoma arises at the lower limbs, as shown in Supplementary Figure 1. Among histological types, nodular melanomas had poorer prognosis. Both the presence of ulceration and mitosis ($p<0.001$ for both) influenced negatively CM patients' survival.

Being Logrank test non-parametrical, continuous variables were categorized as follows:

a: age at diagnosis was categorised into 8 groups as reported in Table 1.

b: 1 for Breslow's depth ≤1 mm; 2 for 1 mm < Breslow's depth ≤2 mm; 3 for 2 mm < Breslow's depth ≤4 mm; 4 for Breslow's depth > 4 mm;

c: 0 for mitotic index=0; 1 for mitotic index<2; 2 for mitotic index ≥ 2

Results on BRAF

During the period of observation 40 patients who recurred were submitted to BRAF mutational status. Results are reported in the following table.

References

1. Grambsch PM, Therneu TM. Proportional hazards tests and diagnostics based on weighted residuals. *Biometrika* 1994; **81:** 515-26.
